# Supplementary figures and images for: Effects of group entitativity on young English-speaking children’s interpretation of inclusive We
Source: PLoS One. 2024 Jul 9;19(7):e0306556. doi: 10.1371/journal.pone.0306556 (PMC11232990; doi:10.1371/journal.pone.0306556)

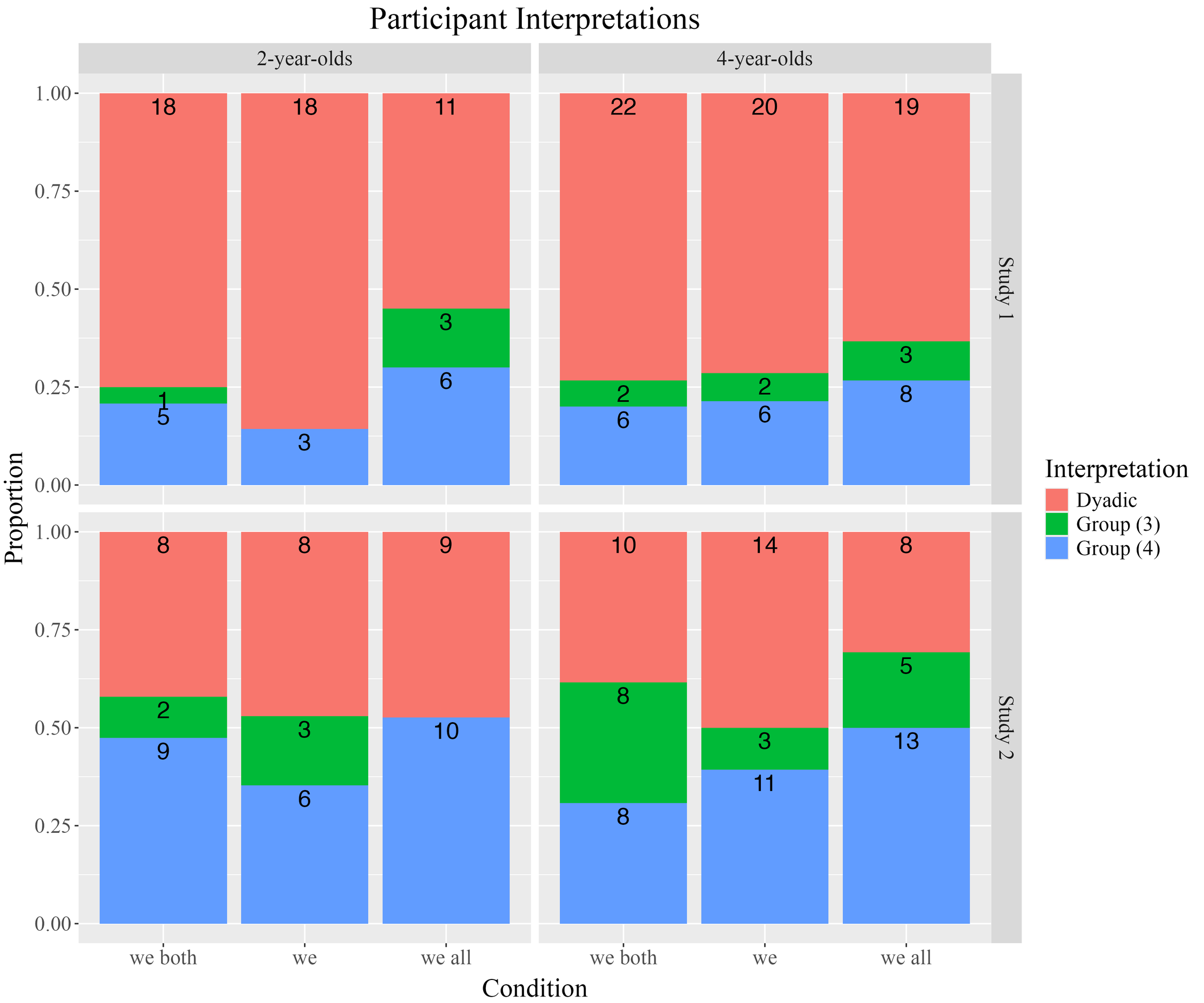

Supplement: S1 Fig — (TIF) [file pone.0306556.s001.tif]

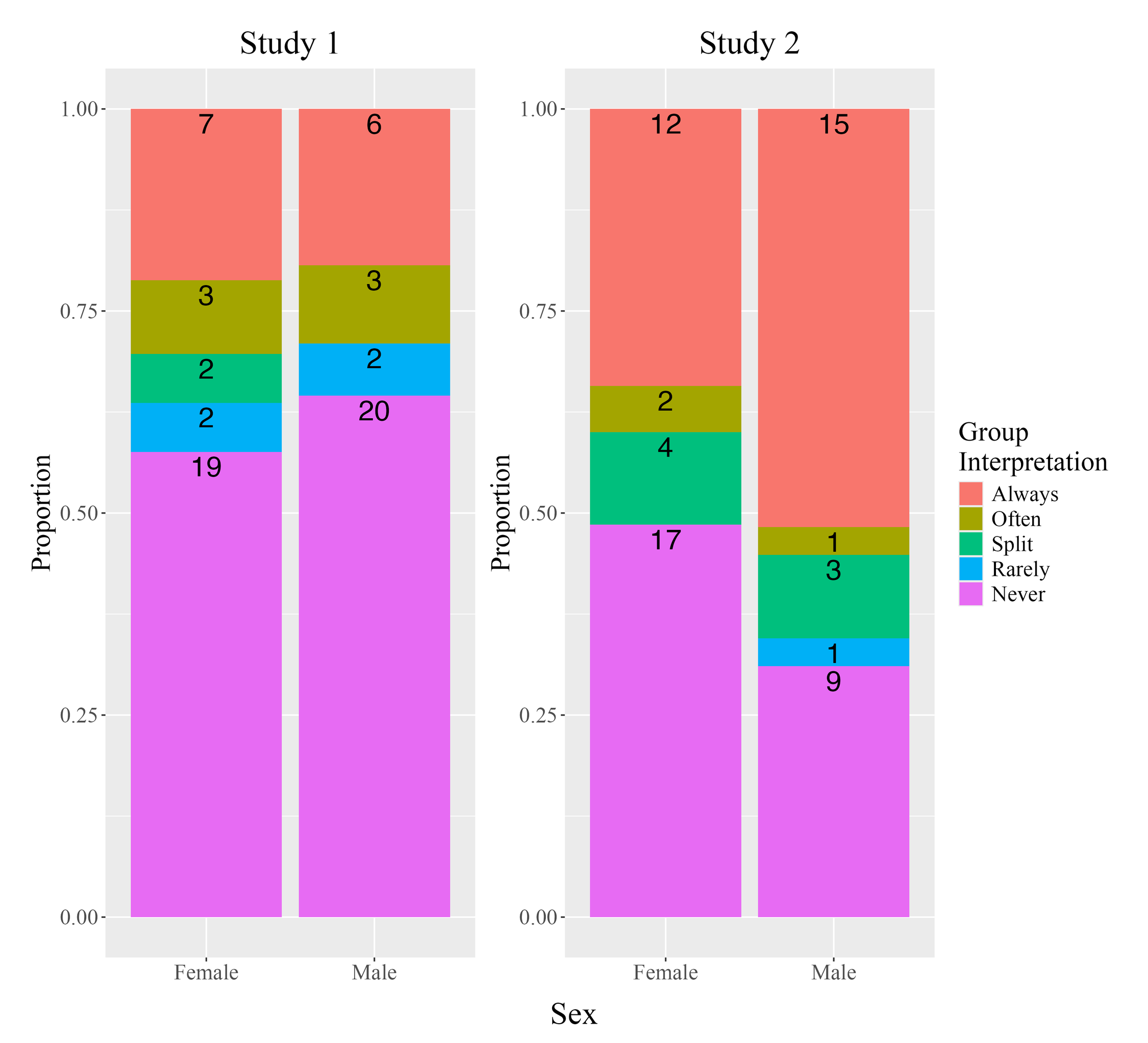

Supplement: S2 Fig — (TIF) [file pone.0306556.s002.tif]

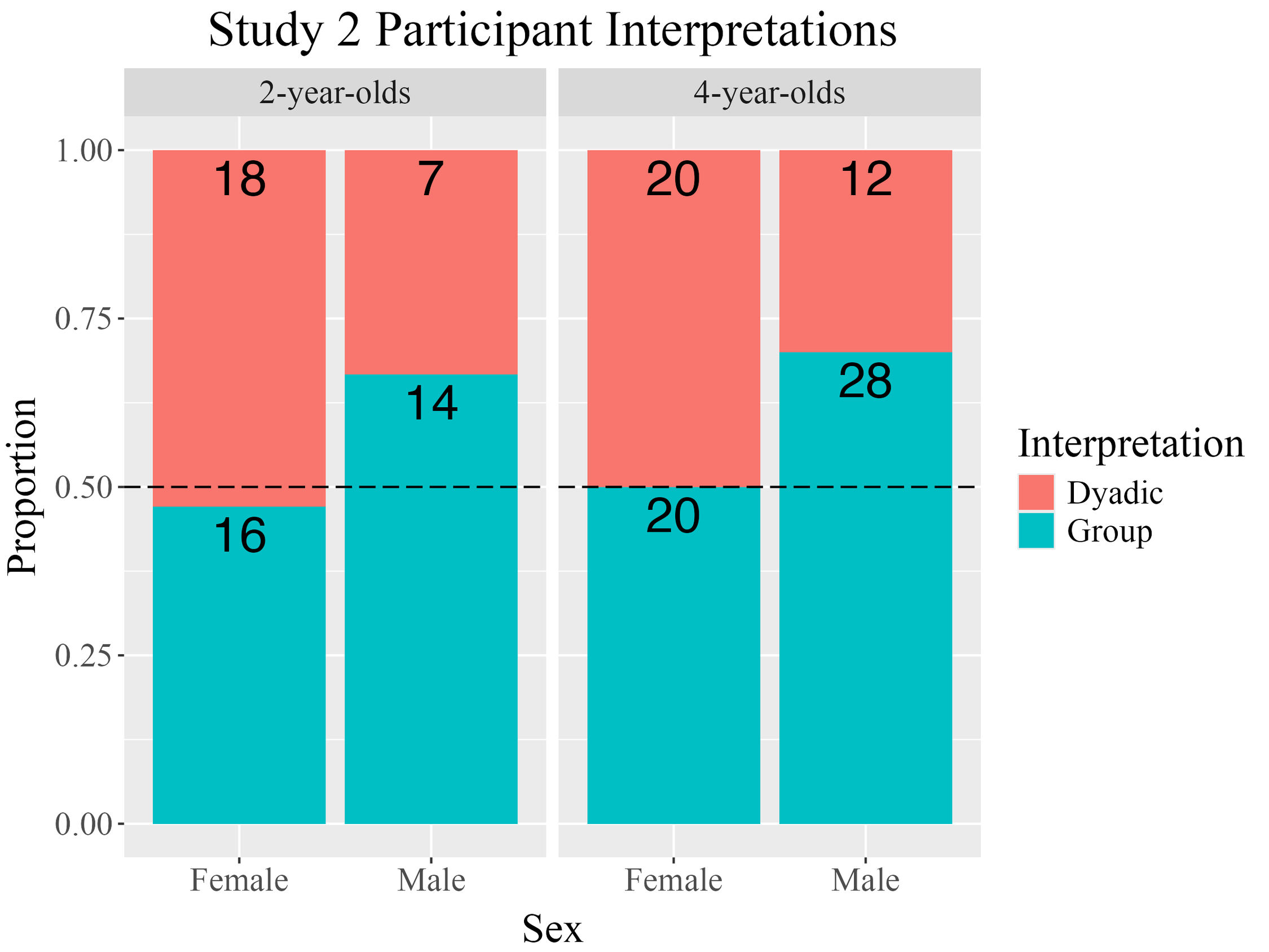

Supplement: S3 Fig — (TIF) [file pone.0306556.s003.tif]

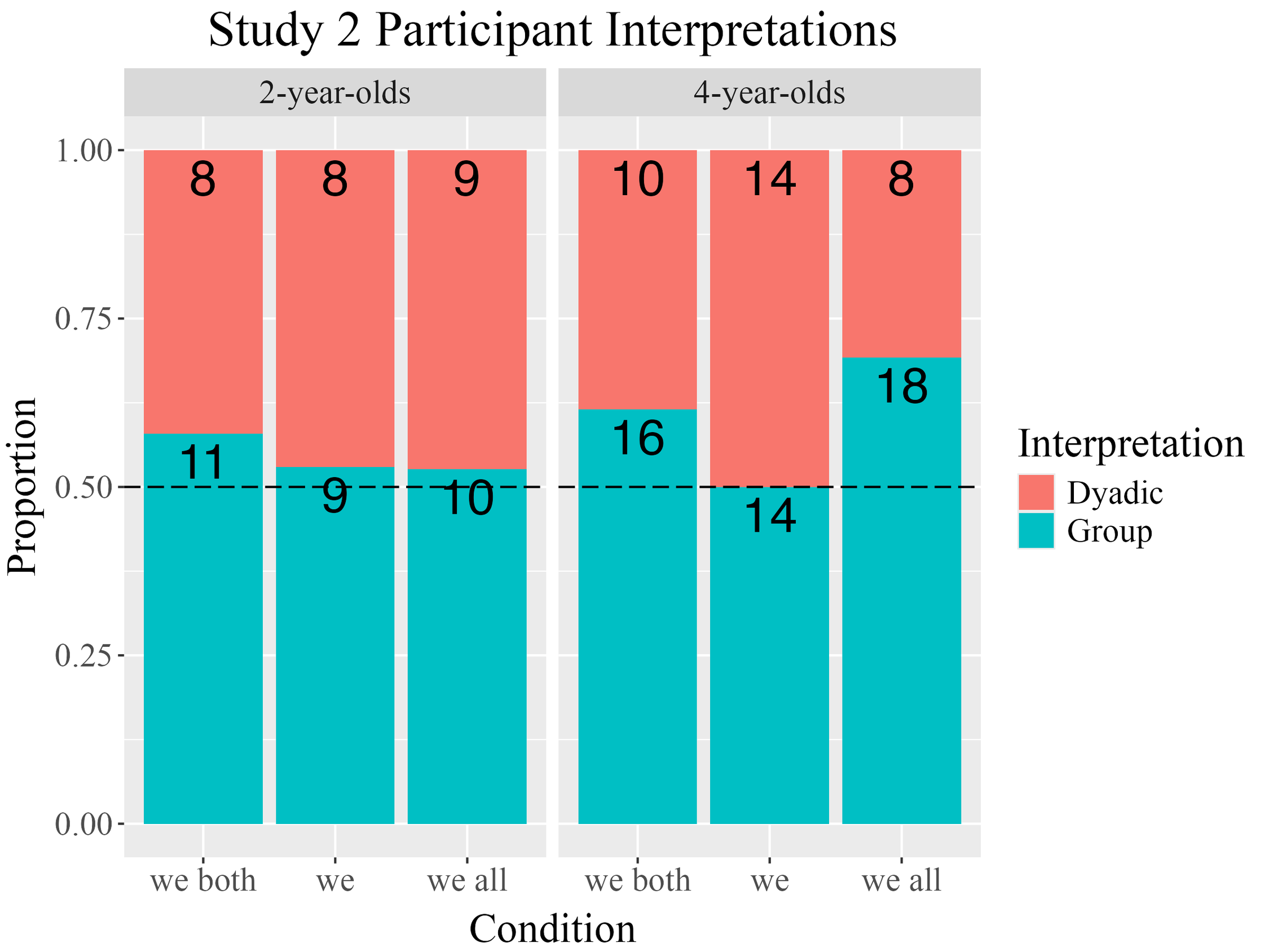

Supplement: S4 Fig — (TIF) [file pone.0306556.s004.tif]
