# Supplementary material for: Effects of group entitativity on young English-speaking children’s interpretation of inclusive We
Source: PLoS One. 2024 Jul 9;19(7):e0306556. doi: 10.1371/journal.pone.0306556 (PMC11232990; doi:10.1371/journal.pone.0306556)
Supplement: S1 Table — (DOCX) [file pone.0306556.s005.docx]

| **Parameter** | **Estimate** | **Error** | **HDI** | **Post. Mass > 0** | **Evid. Strength** |
| --- | --- | --- | --- | --- | --- |
| Intercept | -1.52 | 0.46 | [-2.50, -0.70] | 0.00 | strong |
| Order (we both first) | -0.02 | 0.38 | [-0.76, 0.75] | 0.48 | weak |
| Test trial (2) | 0.33 | 0.33 | [-0.31, 0.97] | 0.84 | weak |
| Test trial (3) | 0.13 | 0.33 | [-0.52, 0.77] | 0.65 | weak |
| Speaker (lion) | -0.12 | 0.34 | [-0.79, 0.53] | 0.37 | weak |
| Speaker (giraffe) | -0.47 | 0.34 | [-1.16, 0.17] | 0.08 | moderate |
| Sex (F) | 0.11 | 0.39 | [-0.64, 0.88] | 0.61 | weak |

**S1 Table**. Posterior parameter estimates of control variables model, Study 1.
