# Supplementary material for: Effects of group entitativity on young English-speaking children’s interpretation of inclusive We
Source: PLoS One. 2024 Jul 9;19(7):e0306556. doi: 10.1371/journal.pone.0306556 (PMC11232990; doi:10.1371/journal.pone.0306556)
Supplement: S2 Table — “Estimate” represents median, “Error” represents 1 SD, “HDI” represents the 95% highest density interval. (DOCX) [file pone.0306556.s006.docx]

| **Analysis** | **Age** | **Condition** | **Parameter** | **Estimate** | **Error** | **HDI** |
| --- | --- | --- | --- | --- | --- | --- |
| First Follow-up | 2- and 4-year-olds | we | Intercept | -1.43 | 0.46 | [-2.37, -0.56] |
|  |  |  | Age Group | 0.45 | 0.52 | [-0.56, 1.48] |
|  |  | we both | Intercept | -1.02 | 0.40 | [-1.84, -0.25] |
|  |  |  | Age Group | 0.05 | 0.48 | [-0.89, 1.00] |
|  |  | we all | Intercept | -0.26 | 0.40 | [-1.05, 0.54] |
|  |  |  | Age Group | -0.21 | 0.47 | [-1.12, 0.71] |
| Second Follow-up | 2-year-olds | NA | Intercept | -1.37 | 0.49 | [-2.40, -0.47] |
|  |  |  | Condition (we both) | 0.06 | 0.54 | [-1.00, 1.11] |
|  |  |  | Condition (we all) | 0.74 | 0.53 | [-0.30, 1.78] |
|  | 4-year-olds |  | Intercept | -1.13 | 0.53 | [-2.20, -0.13] |
|  |  |  | Condition (we both) | -0.16 | 0.52 | [-1.18, 0.86] |
|  |  |  | Condition (we all) | 0.31 | 0.51 | [-0.68, 1.30] |
| Third Follow-up | 2-year-olds | we | Intercept | -1.43 | 0.51 | [-2.48, -0.52] |
|  |  | we both |  | -0.94 | 0.42 | [-1.79, -0.14] |
|  |  | we all |  | -0.16 | 0.41 | [-0.97, 0.62] |
|  | 4-year-olds | we |  | -0.82 | 0.38 | [-1.57, -0.10] |
|  |  | we both |  | -0.89 | 0.38 | [-1.67, -0.18] |
|  |  | we all |  | -0.48 | 0.36 | [-1.22, 0.19] |

**S2 Table**. Marginal posterior distributions of models reported in the three follow-up analyses of Study 1. “Estimate” represents median, “Error” represents 1 SD, “HDI” represents the 95% highest density interval.
