# Supplementary material for: Effects of group entitativity on young English-speaking children’s interpretation of inclusive We
Source: PLoS One. 2024 Jul 9;19(7):e0306556. doi: 10.1371/journal.pone.0306556 (PMC11232990; doi:10.1371/journal.pone.0306556)
Supplement: S3 Table — (DOCX) [file pone.0306556.s007.docx]

| **Parameter** | **Estimate** | **Error** | **HDI** | **Post. Mass > 0** | **Evid. Strength** |
| --- | --- | --- | --- | --- | --- |
| Intercept | 0.37 | 0.4 | [-0.44, 1.17] | 0.83 | weak |
| Order (we both first) | -0.69 | 0.38 | [-1.45, 0.04] | 0.03 | strong |
| Test trial (2) | -0.07 | 0.34 | [-0.75, 0.60] | 0.41 | weak |
| Test trial (3) | 0.49 | 0.35 | [-0.19, 1.21] | 0.92 | moderate |
| Speaker (lion) | -0.19 | 0.34 | [-0.86, 0.47] | 0.29 | weak |
| Speaker (giraffe) | 0.17 | 0.35 | [-0.51, 0.86] | 0.69 | weak |
| Sex (F) | -0.49 | 0.38 | [-1.27, 0.25] | 0.09 | moderate |

**S3 Table**. Posterior parameter estimates of control variables model, Study 2.
