# Supplementary material for: Effects of group entitativity on young English-speaking children’s interpretation of inclusive We
Source: PLoS One. 2024 Jul 9;19(7):e0306556. doi: 10.1371/journal.pone.0306556 (PMC11232990; doi:10.1371/journal.pone.0306556)
Supplement: S4 Table — (DOCX) [file pone.0306556.s008.docx]

| **Parameter** | **Estimate** | **Error** | **HDI** | **Post. Mass > 0** | **Evid. Strength** |
| --- | --- | --- | --- | --- | --- |
| Intercept | -0.06 | 0.57 | [-1.21, 1.04] | 0.46 | weak |
| Order (we both first) | -0.73 | 0.37 | [-1.49, -0.02] | 0.02 | strong |
| Age group (4-year-olds) | 0.20 | 0.58 | [-0.94, 1.36] | 0.63 | weak |
| Condition (we both) | 0.13 | 0.52 | [-0.91, 1.14] | 0.60 | weak |
| Condition (we all) | 0.24 | 0.54 | [-0.80, 1.30] | 0.67 | weak |
| Age group * Condition (we both) | 0.23 | 0.58 | [-0.93, 1.38] | 0.65 | weak |
| Age group * Condition (we all) | 0.57 | 0.60 | [-0.61, 1.77] | 0.83 | weak |

**S4 Table**. Posterior parameters of a model fitted to participants’ Test Trial 1, 2, and 3 data, Study 2.
