# Supplementary material for: Effects of group entitativity on young English-speaking children’s interpretation of inclusive We
Source: PLoS One. 2024 Jul 9;19(7):e0306556. doi: 10.1371/journal.pone.0306556 (PMC11232990; doi:10.1371/journal.pone.0306556)
Supplement: S5 Table — “Estimate” represents median, “Error” represents 1 SD, “HDI” represents the 95% highest density interval. (DOCX) [file pone.0306556.s009.docx]

| Analysis | Age | Condition | Parameter | Estimate | Error | HDI |
| --- | --- | --- | --- | --- | --- | --- |
| First follow-up | 2- and 4-year-olds | we | Intercept | 0.09 | 0.42 | [-0.74, 0.92] |
|  |  |  | Age group (4-year-olds) | -0.08 | 0.48 | [-1.03, 0.87] |
|  |  | we both | Intercept | -0.42 | 0.54 | [-1.50, 0.61] |
|  |  |  | Age group (4-year-olds) | 0.27 | 0.59 | [-0.87, 1.43] |
|  |  | we all | Intercept | 0.69 | 0.53 | [-0.33, 1.77] |
|  |  |  | Age group (4-year-olds) | -0.12 | 0.58 | [-1.27, 1.00] |
| Second follow-up | 2-year-olds | NA | Intercept | 0.08 | 0.42 | [-0.75, 0.90] |
|  |  |  | Condition (we both) | -0.43 | 0.60 | [-1.60, 0.76] |
|  |  |  | Condition (we all) | 0.40 | 0.58 | [-0.73, 1.53] |
|  | 4-year-olds |  | Intercept | 0.09 | 0.45 | [-0.81, 0.99] |
|  |  |  | Condition (we both) | -0.04 | 0.59 | [-1.19, 1.11] |
|  |  |  | Condition (we all) | 0.34 | 0.56 | [-0.78, 1.44] |
| Third follow-up | 2-year-olds | we | Intercept | 0.09 | 0.44 | [-0.77, 0.97] |
|  |  | we both |  | -0.48 | 0.59 | [-1.68, 0.62] |
|  |  | we all |  | 0.62 | 0.58 | [-0.46, 1.78] |
|  | 4-year-olds | we |  | -0.02 | 0.36 | [-0.73, 0.68] |
|  |  | we both |  | 0.00 | 0.55 | [-1.06, 1.11] |
|  |  | we all |  | 0.46 | 0.49 | [-0.46, 1.44] |
| Fourth follow-up | NA | we | Intercept | 0.14 | 0.33 | [-0.52, 0.79] |
|  |  |  | Sex (Female) | -0.19 | 0.31 | [-0.80, 0.43] |
|  |  | we both | Intercept | 0.66 | 0.53 | [-0.32, 1.79] |
|  |  |  | Sex (Female) | -0.75 | 0.46 | [-1.67, 0.13] |
|  |  | we all | Intercept | 0.74 | 0.59 | [-0.30, 2.06] |
|  |  |  | Sex (Female) | -0.39 | 0.48 | [-1.37, 0.55] |

**S5 Table**. Marginal posterior distributions of parameters in models reported in the four follow-up analyses of Study 2. “Estimate” represents median, “Error” represents 1 SD, “HDI” represents the 95% highest density interval.
