# Supplementary material for: Effects of group entitativity on young English-speaking children’s interpretation of inclusive We
Source: PLoS One. 2024 Jul 9;19(7):e0306556. doi: 10.1371/journal.pone.0306556 (PMC11232990; doi:10.1371/journal.pone.0306556)
Supplement: S6 Table — (DOCX) [file pone.0306556.s010.docx]

| **Parameter** | **Estimate** | **Error** | **HDI** | **Post. Mass > 0** | **Evid. Strength** |
| --- | --- | --- | --- | --- | --- |
| Intercept | -0.75 | 0.38 | [-1.54, -0.05] | 0.02 | strong |
| Order (we both first) | -0.46 | 0.35 | [-1.18, 0.22] | 0.09 | moderate |
| Test trial (2) | 0.16 | 0.27 | [-0.36, 0.70] | 0.72 | weak |
| Test trial (3) | 0.36 | 0.27 | [-0.16, 0.91] | 0.91 | moderate |
| Speaker (lion) | -0.16 | 0.28 | [-0.71, 0.38] | 0.28 | weak |
| Speaker (giraffe) | -0.23 | 0.27 | [-0.75, 0.31] | 0.20 | weak |
| Sex (F) | -0.19 | 0.35 | [-0.90, 0.50] | 0.28 | weak |

**S6 Table**. Posterior parameter estimates of control variables model, Comparison of Study 1 and Study 2.
