# Supplementary material for: Effects of group entitativity on young English-speaking children’s interpretation of inclusive We
Source: PLoS One. 2024 Jul 9;19(7):e0306556. doi: 10.1371/journal.pone.0306556 (PMC11232990; doi:10.1371/journal.pone.0306556)
Supplement: S7 Table — (DOCX) [file pone.0306556.s011.docx]

| **Parameter** | **Estimate** | **Error** | **HDI** | **Post. Mass > 0** | **Evid. Strength** |
| --- | --- | --- | --- | --- | --- |
| Intercept | -1.78 | 0.60 | [-2.98, -0.63] | 0.00 | strong |
| Condition (we both) | 0.00 | 0.50 | [-0.97, 0.99] | 0.51 | weak |
| Condition (we all) | 0.61 | 0.50 | [-0.37, 1.59] | 0.89 | weak |
| Study (Study 2) | 0.94 | 0.57 | [-0.21, 2.05] | 0.95 | strong |
| Sex (F) | 0.16 | 0.47 | [-0.75, 1.07] | 0.63 | weak |
| Age group (4-year-olds) | 0.14 | 0.57 | [-1.00, 1.26] | 0.60 | weak |
| Condition (we both) * Study | 0.50 | 0.58 | [-0.64, 1.64] | 0.81 | weak |
| Condition (we all) * Study | 0.16 | 0.57 | [-0.98, 1.29] | 0.61 | weak |
| Condition (we both) * Sex | -0.51 | 0.49 | [-1.47, 0.47] | 0.15 | weak |
| Condition (we all) * Sex | -0.08 | 0.49 | [-1.03, 0.88] | 0.44 | weak |
| Study * Sex | -0.35 | 0.55 | [-1.44, 0.72] | 0.26 | weak |
| Condition (we both) * Age group | 0.02 | 0.55 | [-1.06, 1.12] | 0.52 | weak |
| Condition (we all) * Age group | 0.31 | 0.56 | [-0.80, 1.40] | 0.71 | weak |
| Study * Age group | 0.51 | 0.61 | [-0.67, 1.74] | 0.80 | weak |
| Sex * Age group | -0.16 | 0.56 | [-1.25, 0.93] | 0.39 | weak |
| Condition (we both) * Study * Sex | -0.26 | 0.59 | [-1.41, 0.89] | 0.33 | weak |
| Condition (we all) * Study * Sex | 0.10 | 0.58 | [-1.06, 1.23] | 0.57 | weak |
| Condition (we both) * Study * Age group | 0.24 | 0.63 | [-0.98, 1.47] | 0.64 | weak |
| Condition (we all) * Study * Age group | 0.45 | 0.64 | [-0.80, 1.70] | 0.76 | weak |
| Condition (we both) * Sex * Age group | 0.36 | 0.57 | [-0.77, 1.47] | 0.73 | weak |
| Condition (we all) * Sex * Age group | -0.22 | 0.57 | [-1.33, 0.90] | 0.35 | weak |
| Study * Sex * Age group | -0.28 | 0.62 | [-1.49, 0.94] | 0.32 | weak |
| Condition (we both) * Study * Sex * Age group | 0.28 | 0.64 | [-0.96, 1.52] | 0.67 | weak |
| Condition (we all) * Study * Sex * Age group | -0.22 | 0.64 | [-1.49, 1.00] | 0.37 | weak |

**S7 Table**. Posterior parameters of a model fitted to participants’ Test Trial 1, 2, and 3 data, Comparison of Study 1 and Study 2.
