# Supplementary material for: Effects of group entitativity on young English-speaking children’s interpretation of inclusive We
Source: PLoS One. 2024 Jul 9;19(7):e0306556. doi: 10.1371/journal.pone.0306556 (PMC11232990; doi:10.1371/journal.pone.0306556)
Supplement: S8 Table — (DOCX) [file pone.0306556.s012.docx]

| **Parameter** | **Estimate** | **Error** | **HDI** | **Post. Mass > 0** | **Evid. Strength** |
| --- | --- | --- | --- | --- | --- |
| Intercept | -1.46 | 0.53 | [-2.57, -0.47] | 0.00 | strong |
| Condition (we both) | 0.03 | 0.53 | [-1.01, 1.08] | 0.52 | weak |
| Condition (we all) | 0.49 | 0.56 | [-0.61, 1.57] | 0.81 | weak |
| Study (Study 2) | 0.90 | 0.54 | [-0.15, 1.96] | 0.95 | strong |
| Sex (F) | 0.26 | 0.43 | [-0.59, 1.12] | 0.73 | weak |
| Age group (4-year-olds) | 0.09 | 0.53 | [-0.96, 1.12] | 0.58 | weak |
| Condition (we both) * Study | -0.14 | 0.63 | [-1.38, 1.10] | 0.41 | weak |
| Condition (we all) * Study | 0.37 | 0.62 | [-0.85, 1.59] | 0.72 | weak |
| Condition (we both) * Sex | -0.47 | 0.52 | [-1.46, 0.54] | 0.18 | weak |
| Condition (we all) * Sex | 0.16 | 0.56 | [-0.93, 1.25] | 0.61 | weak |
| Study * Sex | -0.16 | 0.53 | [-1.19, 0.87] | 0.39 | weak |
| Condition (we both) * Age group | -0.07 | 0.61 | [-1.25, 1.13] | 0.45 | weak |
| Condition (we all) * Age group | 0.23 | 0.59 | [-0.93, 1.41] | 0.65 | weak |
| Study * Age group | 0.33 | 0.58 | [-0.80, 1.45] | 0.72 | weak |
| Sex * Age group | -0.12 | 0.51 | [-1.15, 0.89] | 0.41 | weak |
| Condition (we both) * Study * Sex | 0.23 | 0.63 | [-1.02, 1.47] | 0.65 | weak |
| Condition (we all) * Study * Sex | 0.02 | 0.62 | [-1.17, 1.24] | 0.51 | weak |
| Condition (we both) * Study * Age group | 0.09 | 0.67 | [-1.23, 1.41] | 0.56 | weak |
| Condition (we all) * Study * Age group | 0.06 | 0.66 | [-1.20, 1.36] | 0.54 | weak |
| Condition (we both) * Sex * Age group | 0.16 | 0.61 | [-1.04, 1.37] | 0.60 | weak |
| Condition (we all) * Sex * Age group | 0.01 | 0.60 | [-1.17, 1.20] | 0.50 | weak |
| Study * Sex * Age group | -0.26 | 0.59 | [-1.40, 0.90] | 0.33 | weak |
| Condition (we both) * Study * Sex * Age group | 0.45 | 0.66 | [-0.83, 1.74] | 0.75 | weak |
| Condition (we all) * Study * Sex * Age group | -0.29 | 0.67 | [-1.58, 1.03] | 0.33 | weak |

**S8 Table**. Posterior parameters of a model fitted to participants’ Test Trial 1 and 2 data, Comparison of Study 1 and Study 2.
