# Supplementary material for: Effects of group entitativity on young English-speaking children’s interpretation of inclusive We
Source: PLoS One. 2024 Jul 9;19(7):e0306556. doi: 10.1371/journal.pone.0306556 (PMC11232990; doi:10.1371/journal.pone.0306556)
Supplement: S9 Table — (DOCX) [file pone.0306556.s013.docx]

| **Parameter** | **Estimate** | **Error** | **HDI** | **Post. Mass > 0** | **Evid. Strength** |
| --- | --- | --- | --- | --- | --- |
| Intercept | -1.17 | 0.39 | [-1.96, -0.43] | 0.00 | strong |
| Study (Study 2) | 0.94 | 0.46 | [ 0.04, 1.85] | 0.98 | strong |
| Age group (4-year-olds) | 0.21 | 0.44 | [-0.66, 1.08] | 0.68 | weak |
| Study * Age group | 0.05 | 0.51 | [-0.95, 1.07] | 0.54 | weak |
| Intercept | -0.81 | 0.42 | [-1.66, -0.02] | 0.02 | strong |
| Study (Study 2) | 0.25 | 0.52 | [-0.77, 1.28] | 0.68 | weak |
| Age group (4-year-olds) | 0.00 | 0.50 | [-0.99, 1.00] | 0.51 | weak |
| Study * Age group | 0.35 | 0.60 | [-0.84, 1.53] | 0.72 | weak |
| Intercept | -0.06 | 0.53 | [-1.12, 0.99] | 0.46 | weak |
| Study (Study 2) | 0.58 | 0.52 | [-0.44, 1.60] | 0.87 | weak |
| Age group (4-year-olds) | -0.17 | 0.53 | [-1.23, 0.87] | 0.37 | weak |
| Study * Age group | 0.16 | 0.57 | [-0.98, 1.29] | 0.61 | weak |

**S9 Table**. Marginal posterior distributions of parameters in models reported in the follow-up analysis of the Comparison of Study 1 and Study 2.
