# Supplementary material for: Effects of group entitativity on young English-speaking children’s interpretation of inclusive We
Source: PLoS One. 2024 Jul 9;19(7):e0306556. doi: 10.1371/journal.pone.0306556 (PMC11232990; doi:10.1371/journal.pone.0306556)
Supplement: Attachment — Submitted filename: Reviewer 2.pdf [file pone.0306556.s014.pdf]

Review for "Young English-speaking Children's Interpretation of Inclusive We: Effects of Group Entitativity"

This paper presents two coloring-based studies with 2 and 4-year-olds looking at how children younger and older than 3 (a milestone age for changes in groupmindedness) assign colored markers to themself and other puppets (speaker, two others) based on how training transpired and whether the puppet prompting them says sentences with "we", "we both" and "we all". The idea is that "we both" and "we all" should clearly prompt dyads (child participant and speaking puppet) and groups (child, speaking puppet, 1 or 2 other puppets) for marker-giving. "we" by itself is the main question – the authors predict that groupmindedness will play a role. 2-year-olds are before the cluster of changes observed that happen around age 3, so they predict they will be more likely to pick dyads. 4-year-olds are after, so they will pick 3+ sized groups for plain "we". The results are complex, and largely do not accord with the hypothesis – there is no developmental change (main prediction hinges on this). There is overall preference for dyads in the first study, and with multiple training changes to increases the entitativity of the animal puppets (these all made sense), there is a significant increase in group (3 individuals+) assignments of markers. Overall, I unfortunately found this study flawed in terms of stimuli (most notably: the quantifiers "both" and "all" can't be assumed to be known and useful as controls for small children). I don't find that the study results say much about the hypothesis and framing theory (there are too many issues with the task: it could've been simpler and better focused on what children do when they hear "we"). To be publishable it needs major work, and likely further studies. I lay out my concerns below.

_____

Major Concerns:

Lack of developmental linguistic consideration of <u>quantifiers</u> in stimuli ("both" and "all"), in terms of (a) focusing on their effects on responses, and b) reviewing the literature on this rich area of semantic acquisition to assess expectations for 2 and 4-year-olds for each term. Two-year-olds, and even 4-year-olds, are not adult-like in their semantic and pragmatic interpretations of quantifiers, and there is a large body of literature showing that and diving deep into trying to figure out how and why (e.g., Papafragou & Skordos 2016 did a recent-ish overview). I am familiar with that literature and I suspect children should have experience with "all", but I'm more skeptical about "both". This is my most serious concern as it affects design, results, and interpretation. I found a lot of this paper is potentially inadvertently about these two quantifiers and how kids interpret them, but the authors don't acknowledge that. And the proof is in the pudding that these are important in this task, as they didn't work well as controls.

It is surprising to me that these were used as controls. I appreciate that the authors wanted to specify the dyad and group meanings via "both" and "all", but they add many issues. The controls "we both" and "we all" work well for me, probably for adults in general (though see my next concern), but it's a leap to assume they work for children still learning the semantics and pragmatics of these elements.

Better controls, to my mind, would've involved pointing to signal the specific referents (that's how I would distinguish in real life, for clusivity and for size of the group) with no-point in the main test condition, or using numerals like "we two" vs "we three" (likely needing a quick pre-test for number knowledge; a little stodgy sounding though). Or, perhaps best of all - use singular

pronouns and conjunctions, like “me and you”, or “me, you, and Lion”, etc. There are better controls for small children than complex semantic operators.

What if (some) children understand “all” but not “both” - how would that affect the results’ interpretation? *This may be what’s happening with the 2-year-olds in the Pilot.*

Also, even if children understand “all” in an adult-like way as picking out all members of a set, they may differ in what they think the contextually salient set is (i.e. all of what? The domain of quantification. The world often gives options, and this is more or less what the authors are measuring, but how children use the language, which may not be adult-like, could be confounding this). So, even if they get “all” they may still pick a dyad because “all” is still true in a dyad set. This may be what’s happening with 4-year-olds in the Main Study?

In sum, I think the authors need to consider and discuss how word knowledge of quantifiers (or lack thereof) is affecting their results.

Lack of good control condition, and control population:
All the 3 conditions include “we”. So, one way I could recast this study is about interpretations of nothing (plain “we+[nothing]”), vs “both” and “all”. That is what is explicitly varying. I think there are better controls, as I mention above, but I also want a baseline condition without “we” to be able to assess the contribution of “we” and the role of that linguistic prompt. Like what do children do when they’ve had the training, but then there’s just something like “Ok! It’s coloring time!”… do they bias to giving markers to dyads? Everyone? Only themselves? What if it’s the SAME as what they do with “we”? Maybe that fits the hypothesis too? It’s not a linguistic hypothesis per se, just one that interacts with language.

There should be an adult control group. What do adults do with the two tasks? What are their defaults for plain “we”, versus with “both” and “all”? Are they actually as expected? What does adult performance tell us about the stimuli and task? What if adults also have a dyad-bias, what would that mean for the theory?

Where is e.g. Lion looking while talking? If only at the participant (forward) then I suspect that plays a large role in who participants give markers too, and would bias towards Dyadic responses. The Main Study training seems to help override this if this is a constant factor in the two studies. It may be that results relate to how much children rely on that cue to work out what to do, and ages and individuals may differ on that.

Re: gender affects – what was the voicing of the puppets, the experimenter? Could that have an effect? I.e. if male sounding voices, then maybe boys are more likely to include them, and girls to exclude them.

___

Minor Concerns:
“We” is not really “ambiguous” (p4) for referring to the speaker *+1 or more others*, not in the way linguists typically and technically use that word. That is, “we” doesn’t variably refer to dyads or groups, that just never matters for its semantics. Another way of thinking about this - the usage context varies, not the syntax/semantics of the pronoun.

The authors are assuming children already know “we” in an adult-like way (that is, it’s 1st person, plural (where English has no dual, so plural is defined as 2+), and doesn’t distinguish by clusivity). I think the authors need to explicitly discuss prior literature to demonstrate that 2-year-olds know the adult-like meaning of “we” (I’m fairly confident they do? But I’m not sure about that literature and evidence). And then, after establishing that, separate that from the usage contexts of “we” and whether an extra-linguistic bias (dyad vs. group) may affect preference for online choices in the task. (this latter part is clearly done, but the former part not fully).

“We” is not all that infrequent, relatively speaking. I find people use infrequent/rare to mean many different orders of magnitude, and this is uninformative without grounding numbers. Are we talking 1/1000 utterances? 1/10000?

(bottom pg4, top of pg5) I don’t think it’s hard or roundabout to express clusivity in English, we use singular pronouns and conjunctions (me and you, me and them, etc). Or, not English per se, but we use co-speech gestures. That comes very naturally, just pointing back and forth to the addressee and the self, or others and the self. Some mention of gesture, especially pointing out referents, would be warranted, especially as it’s a bit like handing out markers in picking out referents.

Overly complex task introductory language. In the description of the materials and procedure there were many examples with “think” used to introduce indirect assertions and lower certainty of the main proposition, and there were also wish-counterfactuals and conditional counterfactuals (pg. 7). Those are all linguistically and conceptually complex, and I think unnecessary to set up this task. Why were they included? But there was also a mismatch between the prose I’m referring to and the sample item (Figure 1) (I think because Figure 1 shows only a trial set-up, the prose is also the overall set-up?). Assuming the sample item is correct for what the children heard, this is a minor concern to make the prose match. If the children did hear counterfactuals and modals, that needs to be justified as 2-year-olds in particular are not adult-like in understanding those. Why not just “Who should I give the blocks to?” “think” was especially odd in the prompts like on pg. 9, “Great! We can color! I think we can color! We can color together!” – here the use of “think” reads to me (a native speaker) like the speaker is uncertain we can (perhaps we’re not allowed?).

How does entitativity development relate to number and set reasoning development? (i.e., how social is this, or does it also relate to inanimates). It would be interesting to cover this.

Individual results? The experiment wasn’t really designed to get much data from individuals, but there are participants who provide 3 data points for the studies. But, it’s clear what we expect given hypotheses– dyad for “both”, group for “all” and dyad for 2yos “we”/group for 4yos “we”. If a child knows the meanings, especially of the “both”/”all” controls, that’s what they should do (for “we” more options). Looking at this could be informative? Not sure, worth looking.

(p35, concluding paragraph) The interpretations aren’t what I would call “spontaneous”, as they are occurring in a highly constrained task with a training period. “Online” perhaps? “Spontaneous” is typically only used for non-experimental uses or interpretations, happening in e.g., home contexts.
