# Supplementary material for: Effects of group entitativity on young English-speaking children’s interpretation of inclusive We
Source: PLoS One. 2024 Jul 9;19(7):e0306556. doi: 10.1371/journal.pone.0306556 (PMC11232990; doi:10.1371/journal.pone.0306556)
Supplement: Attachment — Submitted filename: rev1_Response to Reviewers.docx [file pone.0306556.s015.docx]

**Reviewer 1**

This paper reports the results of two virtual experiments examining the interpretation of the inclusive pronoun “we” by 2- and 4-year-olds. The two experiments differed in the context of the task. In the first experiment, the partners (and potential referents of the pronoun “we”) appeared like an aggregation of individuals, whereas in the second, they were presented as a collaborative group. The results showed that children more often interpret “we” as referring to a group in a collaborative group context (second experiment) than when it was not the case (first experiment) but, and it is a weakness of the paper, it is the only reliable result of the study. However, the paper is clear and well-written, the study is undertaken in a careful manner and the data are handled appropriately.

Minor comments:

Naming the two experiments “pilot experiment” and “main experiment” respectively is not really justified, so why not just name them Experiment 1 and Experiment 2.

We thank the Reviewer for their attention to our manuscript and for their thoughtful commentary and feedback on the original draft of the manuscript.

All uses of Pilot Study and Main Study have been replaced with Study 1 and Study 2, in the Main Text, Supplementary Material, and all tables and figures.

It is rather surprising that the authors refer to the variable sex when they are apparently referring to the gender of the participants. Unless they are referring solely to biological and physiological differences between males and females, the variables sex and gender are at least confounded here. This point needs to be clarified.

We were referring to the sex of the participants because we asked participants’ caregivers only what sex their child is. Gender was never inquired so we do not refer to it.

The methodology sections are a little confusing to read. In the first experiment, in the participants section, it is mentioned that seven participants were excluded and then, in the coding section, it is mentioned that 60 trials were excluded, which seems to take into account the trials of the participants excluded above. Why not mention here only the trials excluded from the trials of the 64 participants retained for the study? Same comment for the second experiment.

After rereading this portion of the manuscript, we agreed with the Reviewer’s suggestion. We have rephrased the manuscript in line with the Reviewer’s suggestion (revisions on pp. 11 and 22).

From the text of the first study, the original text read: “In the final analysis, 153 trials were included (*N* = 64 participants) and 60 trials were excluded (*N* = 33 participants). Of the 153 included trials, 38 participants contributed 3 trials (114 trials), 13 contributed 2 trials (26 trials), and 13 contributed 1 trial (13 trials). Of the 60 excluded trials, 7 participants contributed 3 trials (21 trials), 13 contributed 2 trials (26 trials), and 13 contributed 1 trial (13 trials).”

The revised text (p. 11) now states: “In the final analysis, 153 trials were included (*N* = 64 participants) and 39 trials were excluded (*N* = 26 of the 64 participants). Of the 153 included trials, 38 participants contributed 3 trials (114 trials), 13 contributed 2 trials (26 trials), and 13 contributed 1 trial (13 trials).”

From the text of the second study, the original text read: “In the final analysis, 135 trials were included (*N* = 64 participants) and 129 trials were excluded (*N* = 62 participants). Of the 135 trials included in the analyses reported, below, 26 participants contributed 3 trials (78 trials), 19 contributed 2 trials (38 trials), and 19 contributed 1 trial (19 trials). Of the 129 excluded trials, 24 participants contributed 3 trials (72 trials), 19 contributed 2 trials (38 trials), and 19 contributed 1 trial (19 trials).”

The revised text (p. 22) now states: “In the final analysis, 135 trials were included (*N* = 64 participants) and 57 trials were excluded (*N* = 38 participants). Of the 135 trials included in the analyses reported, below, 26 participants contributed 3 trials (78 trials), 19 contributed 2 trials (38 trials), and 19 contributed 1 trial (19 trials).”

Still regarding excluded trials, “Of the 60 excluded 9 trials, 7 participants contributed 3 trials (21 trials), 13 contributed 2 trials (26 trials), and 13contributed 1 trial (13 trials)” (p.11). Is this information really important? And if so, what does it contribute? If not, I suggest dropping this information. Same comment for the second experiment.

After rereading these portions of the manuscript, we agree with the Reviewer’s suggestion. We have removed these lines in the revised manuscript (please see previous response).

Please do not use E for experimenter or put it in brackets when first mentioned. Idem for P for participant.

The first use of E for experimenter was placed in brackets (p. 7). Additionally, P for participant has been replaced with “participant” in Figure 1 (p. 10); this was the only location in the manuscript in which this form was used.

I'm not familiar with Bayesian models (and I guess I'm not the only one), how were the priors chosen (p.12)?

We respond in two parts. First, we note that we have revised the manuscript text by adding the following text: “Priors were chosen based on general domain knowledge and an understanding of the response distribution defined by the link function so as to be “weakly informative” about the most likely outcomes. This strategy is considered best practice in Bayesian modeling (Gelman et al., 2013; Lemoine, 2019; Schad et al., 2021) and has been used in previous developmental psychological analyses (Vasil et al., forthcoming; Vasil & Tomasello, 2022),” (p. 13).

Second, we elaborate on the revised text. As noted, priors were chosen based on domain knowledge and an understanding of the response distribution defined by the link function so as to be “weakly informative” about the posterior distribution. This is considered best practice in Bayesian modeling and has been used in previous analyses of development psychological experiments. For example, general domain knowledge (e.g., from previous child development studies) suggests that, though not impossible, it is unlikely that a manipulation will switch binary choice behavior from 99% response X to 99% response Y. This intuition can be formalized as a (prior) probability distribution over the parameter space of the response distribution with maximum likelihood on 0 (i.e., that signals no effect, chance, etc.). When the logit function is used to link the predictor and response distributions, values greater than 1.5 or less than -1.5 (i.e., plus/minus two standard deviations of the SD of the prior distribution) are considered *a priori* highly unlikely, though not impossible. That is, predictor odds ratio values that have an absolute value greater than 1.5 lie outside the set of 95% *a priori* most likely odds ratios. Heuristically, the logic behind Bayesian methods can be understood as generalizing frequentist methods from inference about point estimates to inference about the distribution of point estimates (Gelman et al., 2020).

The same logic used to define the prior distributions of the predictor variables was applied to definitions of the prior distributions over the other parameters, including the intercept and hierarchical variance parameters.

As underlined by the authors in the general discussion (p.32), the variable sex/gender was not purposely manipulated (in both experiments, there were far fewer males than females) and the observed effects of sex/gender were statistically weak. The effects of this variable are therefore not worth mentioning in the abstract and in the final conclusion (p.35).

We agree with the Reviewer that associations with sex should not be mentioned in the abstract and final conclusion. Accordingly, we have removed mention of sex in the abstract and in the final conclusion (p. 36). We have additionally removed what we considered to be redundant text (p. 20). That text originally read: “There was little evidence for associations between other predictors and participant interpretations; for example, both sexes favored dyadic interpretations.” The revised text excludes the portion of text after the semicolon.

Moreover, we have fixed an error in reporting of sex demographics of the comparison study. Originally, it was stated that 11 2-year-olds males and 21 4-year-old males were included in the Comparison of Study 1 and Study 2 analyses. These numbers were erroneously imported from (correct) reporting of those same numbers in the participants section of Study 2 (p. 21). The revised manuscript corrects this error by reporting the added values from studies 1 and 2. Thus, there are reported to be 29 2-year-old males and 34 4-year-old males in the comparison analyses (p. 28).

**References**

Gelman, A., Carlin, J., Stern, H., Dunson, D., Vehtari, A., & Rubin, D. (2013). *Bayesian Data Analysis* (3rd ed.). CRC Press/Taylor & Francis.

Gelman, A., Hill, J., & Vehtari, A. (2020). *Regression and Other Stories*. Cambridge University Press.

Lemoine, N. P. (2019). Moving beyond noninformative priors: Why and how to choose weakly informative priors in Bayesian analyses. *Oikos*, *128*(7), 912–928. https://doi.org/10.1111/oik.05985

Schad, D. J., Betancourt, M., & Vasishth, S. (2021). Toward a principled Bayesian workflow in cognitive science. *Psychological Methods*, *26*(1), 103–126. https://doi.org/10.1037/met0000275

Vasil, J., Price, D., & Tomasello, M. (forthcoming). Thought and Language: Effects of Groupmindedness on Young Children’s Interpretation of We. *Child Development*.

Vasil, J., & Tomasello, M. (2022). Effects of “we”-framing on young children’s commitment, sharing, and helping. *Journal of Experimental Child Psychology*, *214*, 105278. https://doi.org/10.1016/j.jecp.2021.105278

**Reviewer 2**

The data doesn't support all of the conclusions drawn. The results are potentially confounded in a few different ways (detailed in my review) and yet the authors - while very open and honest about what the stats show - still conclude mostly with the hypothesis and don't consider reasonable alternatives that may explain their results (which were mostly unexpected relative to the hypothesis)

We thank the Reviewer for their attention to our manuscript and for their thoughtful commentary and feedback on the original draft.

As the only positive conclusion we drew from the study was the relationship between group entitativity (Study 1 vs. Study 2) and interpretation of *we*, *we both*, and *we all*, we understood the Reviewer’s concern to pertain to this conclusion. In the original manuscript, we concluded that children’s interpretation of *we*, but of neither *we both* nor *we all*, was influenced by group entitativity (with “entitative” groups pulling more for group rather than dyadic interpretations). Moreover, we presented a subsequent paragraph that elaborated an interpretation of this conclusion.

After carefully rereading and reconsidering these portions of text, we agree that our conclusion was not fully supported by the evidence. In light of other Reviewer comments (below), we have revised this portion of text. The subsequent interpretation paragraph, noted above, was removed and replaced with a single sentence that more tentatively suggests the same interpretation. Moreover, this is followed by a new paragraph that relates our revised, more careful conclusion to limitations in the study design noted by the Reviewer. Specifically, in the revised text, we now state (p. 34):

“One interpretation of this pattern is that participants who saw the puppets as a group entity (in Study 2) relied more heavily on nonlinguistic contextual cues to resolve pragmatic ambiguity about the intended referent of *we* compared to *we both* or *we all* (Vasil, 2023). Importantly, however, one limitation of the present study limits how far we wish to push any specific interpretation of this result, at present.

Perhaps the main limitation of the present research was the materials used in the present study. Future research might consider using materials different than those used in this research. The materials used in the present studies required children to know how to interpret uses of the phrases *we both* and *we all*, or at least of the quantifiers contained in those phrases. However, the frequency of these forms in young children’s input is unknown. Thus, it is possible that our failure to find the predicted results owed, at least in part, to participants’ having not known the phrases *we both* and *we all*, or at least their constituent quantifiers. In addition, one might view these terms as inviting children to draw scalar implicatures about speaker-intended meaning, something with which young children struggle (Papafragou & Skordos, 2016). Moreover, young children struggle to appropriately understand uses of universal quantifiers, like *all*, even in situations in which scalar implicatures do not have to be drawn (Brooks & Braine, 1996). Taken together, the present studies demonstrate that young children’s interpretation of bare *we* is impacted by the apparent entitativity of the group of which the speaker is part. This much is clear. However, it is unclear why this is the case. Is it because young children rely more on relevant nonlinguistic cues to resolve referential ambiguity for *we* compared to *we both* or *we all*, or because they simply do not know these forms?”

**Lack of developmental linguistic consideration of quantifiers in stimuli (“both” and “all”)**

The Reviewer suggests several insightful limitations of the present research. After carefully rereading the original manuscript, we have decided to introduce discussion of some of these limitations in the revised manuscript (reproduced above from p. 34 of the revised manuscript). We add only that it is possible that children, or some children, learn the use of such quantifiers as *all* and *both* in the forms *we all* and *we both* not as analyzed, constituent parts of quantified first-person plurals (e.g., [*we*] [*both*]) but, instead, as unanalyzed, frozen phrases (e.g., [*we both*]). We have alerted readers to both possibilities in the revised text.

**Lack of good control condition, and control population.**

We first respond to the Reviewer’s comment about a no-*we­* control condition before responding to their comment about an adult control population. The Reviewer’s point about a comparison between a hypothetical no-*we­* condition and the *we* condition is well taken. We agree that it would be an interesting comparison. However, while our focus in the manuscript was on the relationship between group-mindedness and language development, we do not claim that such effects of group-mindedness are unique to language. In fact, we believe that these effects are not unique to language, and evidence for this is reviewed in Introduction. However, nothing in our research question hangs on how group-mindedness influences other facets of psychological development. Therefore, we consider a no-*we* control condition to be outside the scope of the study. We suggest that, if we changed the research question, or retained the same research question but claimed that the predicted effects were unique to language, then, in that case, we agree that a no-*we* control condition would be needed.

We agree with the Reviewer that an adult sample would be an interesting comparison with the current sample. A suggestion for this has been added to the revised manuscript (p. 36). In particular, it would be useful to ensure that the referring situations used in the present study actually invite the interpretation of *we both* and *we all* that we presuppose. Nonetheless, we do emphasize that it seems especially unlikely that *we both* is interpreted as anything other than dyadic by adults (though, this should be empirically examined). Moreover, it seems especially unlikely that *we all* is interpreted as anything other than a group interpretation (or, perhaps more accurately, a non-dyadic interpretation – or else, why not just say *we both*)? This latter suggestion might be seen as relying on processes of statistical preemption, as emphasized by Adele Goldberg and others and of which young children are capable (reviewed in Tomasello 2003, *Constructing a Language*). In any case, we agree that future research should examine an adult comparison group, to empirically examine the presuppositions that underlie the control conditions in the present studies.

**Where is e.g. Lion looking while talking?**

All puppets were facing forward while talking (looking “at” the child). Thus, we could not determine the effect of this variable on children’s interpretations.

We agree with the Reviewer that, at least in Study 1, this may have impacted children’s interpretations by biasing them to favor dyadic responses. Indeed, this represents a degree of ecological invalidity, as speakers (by our anecdotal experience) tend to look at not only a single listener when uttering *we* but, also, other listeners when those other listeners are present and included in the referent of *we*. However, we were concerned that this might bias participants differently depending on the onscreen location of the speaker puppet (e.g., if the speaker puppet was the leftmost puppet, they could “look” at the puppet nearest them, while the middle puppet would have to look at the either one of the two puppets nearest them or both puppets, etc.). Thus, we opted to control this variable by having all puppets look only at participants. We view this as an important variable to examine – and one that would increase the ecological validity of the present studies – but which is outside the scope of the present research.

**Re: gender affects – what was the voicing of the puppets, the experimenter?**

The giraffe was voiced by a male native English speaker and the other two puppets were voiced by female native English speakers. This information has been added in the revised manuscript on page 7.

We examined the relationship between the sex of the (voice of the) puppet and participants’ responses overall and by participant sex. Considering the data from both studies, combined, there were no reliable relationships according to the descriptive and inferential statistics we computed.

Specifically, female participants chose dyadic interpretations of female-voiced speaker puppets on 41 trials and males did so on 32 trials. In contrast, female participants chose dyadic interpretations of male-voiced speaker puppets on 19 trials and males did so on 20 trials.

Turning to group interpretations, female participants chose group interpretations of female-voiced speaker puppets on 18 trials and males did so on 20 trials. In contrast, female participants chose group interpretations of male-voiced speaker puppets on 9 trials and males did so on 9 trials.

Three models were fitted to the relevant data. First, logistic regression with predictors of participant sex, study, and speaker puppet sex (up to three-way interaction) showed no reliable association with participants’ interpretations. A pair of additional logistic regressions with predictors of participant sex and study (and their interaction) found no reliable associations when looking only at female-voiced speaker puppets (second model) or the male-voiced speaker puppet (third model).

**“We” is not really “ambiguous” (p4) for referring…”**

We were using “ambiguity” in the way typically used in the developmental research with which we were familiar (citations in Introduction), and not in the truth-functional meaning employed by generative linguists (literature that we did not cite). Regardless, we have added the following text: “Note that this is a kind of “pragmatic” ambiguity in referential intentions (also, e.g., Bohn et al., 2020), and not semantic ambiguity (i.e., as “ambiguity” is understood to pertain to truth conditions). Nonetheless, resolution of this ambiguity is psychologically important for listeners because the former referential intention warrants a dyadic interpretation, whereas the latter warrants a group interpretation,” (p. 4). Where it might be useful, we have replaced “ambiguity” with “pragmatic ambiguity” or the like throughout the abstract and introduction.

**“The authors are assuming children already…”**

We have added the following text to the Introduction: “Importantly, 2- and 4-year-olds displayed an adult-like understanding of the first-person semantics of *we* by reliably including the speaker as one of the individuals referred to by use of *we* (number was controlled, which precluded investigating children’s understanding of the plural semantics of *we*),” (p. 4).

**“We” is not all that infrequent…”**

We have added the following text to the Introduction: “For example, Vasil et al. (2023) found that *we* and other first-person plural personal, possessive, and reflexive pronouns constitute about 6% of their mothers’ spontaneous pronouns and occur in only about 3% of their mothers’ spontaneous utterances,” (p. 5).

**“(bottom pg4, top of pg5) I don’t think it’s hard or…”**

We had intended the original use of “roundabout” to mean “multiword.” We have changed the wording accordingly to “multiword constructions,” (p. 5).

We were uncertain about how to discuss gesture in the suggested context. We agree that handing out markers may have functional similarities to pointing in indicating intended referents. However, it seems to us like the Reviewer suggested that we discuss gesture as a means for speaker puppets to indicate their referential intent, on analogy with distributing markers to their intended referents. But participants were the ones who distributed the markers, and so it was unclear to us how to relate this to gesture within the scope of the suggestion.

**“Overly complex task introductory…”**

We apologize for the errors in the quotations in Figure 1. The revised Figure 1 (p. 11) has these errors amended so that the quotations in the figure align verbatim with those in the text. The text in the manuscript that included propositional attitude verbs, conditionals, and counterfactuals was the correct text; the text in the original Figure 1 was incorrect.

We agree with the Reviewer that the language used to structure the task for participants could have been simpler. The language was chosen to be engaging for participants while allowing use of the key (manipulated) linguistic forms as much as possible without the discourse seeming overly stilted in the online setting, or too repetitive and thus boring. We kindly note two important points. First, this worrisome language in question was identical between conditions, and so cannot be used to account for any effects between conditions or studies. Second, we suggest splitting the worrisome language into language that the experiment (E) used and the speaker puppet.

E’s use of such phrases as “I think…” is not of particular concern to us (although this should be simplified in future research) because children understood the task. That is, E’s use of these forms would be worrisome to us if there was some suggestion that children did not understand the task as introduced by E. However, all children understood that they were coloring with the puppets (as indicated by their assent to questions about whether wanted to color with the puppets) and understood via the warmup that the task involved directing E to distribute items to the puppets.

Thus, our main concern is with the speaker puppet’s relatively complicated language. We agree with the Reviewer that this language is somewhat odd. Thus, we have suggested that the linguistic materials be simplified in future research (p. 34). However, we point out the substantial language surrounding that worrisome portions in which the key (manipulated) forms were used in simple sentences that lacked relatives, conditionals, counterfactuals, etc. Moreover, we suggest that, were there age- or experience-related processing difficulties for the 2-year-olds, then this should have caused 2-year-olds to respond systematically differently than 4-year-olds in all conditions. However, this was not the case.

**“How does entitativity development…”**

We agree that children’s numerosity may have relevance to the present studies. However, a proper discussion of this was well outside the scope of the intended study. Thus, we have added the following text as a future directions point (p. 36). “Finally, future research might investigate potential relations between children’s interpretation of *we* and their understanding of the symbolic representation of numerosity. Research indicates that, at around 3 years of age, powerful skills emerge that enable children to link linguistic forms that symbolize cardinality with the number of elements in a set (Wynn, 1990), and this has consequences for their ability to count to numbers larger than three.”

**“Individual results? The experiment…”**

We analyzed participant-level data for all participants who contributed 3 trials (*N* = 64 participants across studies 1 and 2). Specifically, we grouped children into those who responded the same to *we all* and *we both* as to *we* (“same-same”), e.g., by picking a dyadic interpretation for all of them; those who responded the same to *we all* and *we* and differently to *we both* (“same-different”), e.g., by picking group interpretations for *we all* and *we all* and a dyadic interpretation for *we both*; those who responded the same to *we both* and *we* and differently to *we all* (“different-same”); and those who responded one way to *we* and differently to *we all* and *we both* (“different-different”), e.g., by choosing a dyadic interpretation for *we* and a group interpretation for *we both* and *we all*. Using this participant-level grouping, we found a similar pattern of descriptive results documented in the group-level analyses presented in the manuscript. Specifically, the clearest association emerged between studies, rather than with children’s age.

Most children in both studies were grouped into the “same-same” category. Specifically, 28 children were same-same in Study 1 (out of 38 children) and 22 were same-same in Study 2 (out of 26 children). Of the 28 same-same children in Study 1, 23 chose dyadic interpretations for *we*, *we both*, and *we all*; 5 chose group interpretations for all three forms. In contrast, of the 22 same-same children in Study 2, 14 chose group interpretations for all three forms, while 8 chose dyadic interpretations. Like the group-level analyses, these participant-level analyses suggest that the main drive of children’s interpretations was the entitativity of the group of puppets.

**“(p35, concluding paragraph) The interpretations aren’t what…”**

We have removed all such uses of “spontaneous” from the manuscript.
