# Supplementary material for: Effects of group entitativity on young English-speaking children’s interpretation of inclusive We
Source: PLoS One. 2024 Jul 9;19(7):e0306556. doi: 10.1371/journal.pone.0306556 (PMC11232990; doi:10.1371/journal.pone.0306556)
Supplement: Attachment — Submitted filename: rev2_Response to Reviewers.docx [file pone.0306556.s016.docx]

**Reviewer**

We thank the Reviewer for their attention to the manuscript, kind remarks, and helpful comments. We have made minor revisions to the manuscript following their suggestions. Our responses are provided in green text, below, following each comment.

Reviewer #3: Thank you for allowing me the opportunity to read this manuscript. The paper is very clearly and well written, and the methods were obviously very carefully designed. The following “review” is different from a review I normally write. It is instead more like a musing on the current findings (which is a signal that it is an interesting paper!). I do think recruiting some adult participants to engage in the tasks that children did (or some modification of them that is more appropriate for adults) would help disambiguate whether results tell us something about development (only) and/or whether some aspects of study design and linguistic stimuli drive interpretations in some unexpected ways (for all English speakers, regardless of age). The authors suggest doing this in the discussion with lab members, but I would caution against including any adult participants who know anything about the current study and its aims. I motivate this idea in detail in the following:

When I read the abstract for the study and initially scanned the methods, I anticipated three groups to which “we” would refer:
1. The speaker puppet and interlocutor (child); the “dyad” interpretation, included in this paper
2. The speaker puppet and interlocutor (child), along with an/some additional puppet(s); the “group” interpretation, referred to in the paper
3. The speaker puppet and the other puppets; not included in the paper
The interpretation of 3 as a group is not discussed as a possibility in this paper, but when I did my initial reading, I actually thought that that group seemed like the most likely and salient interpretation for “we,” since all three puppets are: 1) on the same side of the computer; 2) in the same physical space; 3) have important attributes in common – they are all animals and puppets! I was worried that the dominance of the puppet group might confuse findings.

Happily, upon reading the methods more carefully, I learned that the researchers designed their methods so that the child always considered themselves a part of the group. Correspondingly, results seem to indicate that the careful design of the study, along with warmup trials and preliminary discourse (e.g., making sure the child had markers, too) encouraged children to consider themselves a member of “we.”

We have revised the Abstract to the manuscript to make our intentions clearer. Specifically, in the revised manuscript we have added text (Abstract) that mentions that “This article reports the results of two online experiments that investigated children’s understanding of inclusive *we*, in which the child-listener is part of the intended referent of *we*.” Additionally, we have added text that states that “Participants had their own markers and had to choose to how many partners to distribute (virtual) markers.”

However, I do think the confusing findings related to “we both” may be (at least partially) explained by the salience of the puppet group. “We both” is an odd expression. Since first reading this paper, I’ve been thinking about how I use that phrase. It is harder to think of situations when I would use “we both” to request than it is when I would use it to describe past or future events (“We both felt that way!” or “tomorrow we both are going to go vote”). Importantly, the situations in which I would use “we both” to request/command are ones when I’m emphasizing that another person should do something with me (i.e., I’m emphasizing the “we” by using “both”, e.g., “no, we BOTH need to go vote tomorrow”) rather than clarifying that I mean a dyadic group vs a larger group. To specify a dyad (vs a group), I would say “you and I”. Not “we both”.

We have revised the Discussion Section to include discussion of this possibility. In the revised Discussion Section (pp. 34-5), we note that “Relatedly, it is plausible that the departure children’s interpretation of *we both* and *we all* from predictions does not have to do with their inexperience with these forms. Instead (or in addition), children’s conceptual skills may be involved. Checking this requires additional control conditions that enable comparison of children’s interpretation of *we* and its quantified forms to that of forms like *you and I*, or *you, me, and Lion*. If children’s interpretations of those forms aligns with their interpretations of *we both* and *we all*, as in the present studies, then this may suggest that children’s conceptual skills are implicated in explaining the results of the present studies.”

Returning to the current paradigm, if I were on a zoom call with a group of people (where the other people were all in the same space, like the puppets are in the current study), and one of them said to me “we both can take notes,” I believe I would interpret “we both” as referring to the speaker and one other member of the group of people in the same physical space, on the other side of the screen. If the speaker wanted to specify that she meant to include me and herself, I would expect her to say, “you and I can take notes” (while looking at me – I see a previous reviewer talked about the role of gaze).

Now, again, I know that the current paradigms do a lot to ensure that the person on the other side of the zoom call (the child in this case) feels like they are always a member of “we”, so this may, again, override the saliency of the fact that the puppets are all in the same room.

We have revised the Discussion Section to include discussion of this possibility. In the revised Discussion Section (p. 36), we note that “It is possible that the predicted pattern for children is, in fact, not the actual one. A check on this is to conduct an in person analogue of the present studies. This is important because the pragmatics of the current procedure was stilted. Children had to infer that they were part of an intended, first-person plural referent that included individuals who were together, onscreen; children had to infer that they were part of a group with individuals displayed onscreen. This situation is not representative of most children’s naturalistic interactions, which are primarily in person and not online. It will be important, too, to include adults as a control group. This is necessary to ensure that the expected adult interpretations of *we both* and *we all* do, in fact, obtain.”

Still, this makes me wish that the paradigm was tested on adults. If other adults interpreted sentences the way I (think) I would, they should show a tendency to give markers to two puppets for “we both” and to three puppets for “we all.” Would it be possible to run some version of this paradigm on a few adults, who are naïve to the study’s goals? I do think that their interpretations would be helpful in understanding children’s responses.

We have revised the Discussion Section to include discussion of the importance of testing adults in future research using the present paradigm. Specifically, in the revised Discussion Section (p. 36), we note that “It will be important, too, to include adults as a control group. This is necessary to ensure that the expected adult interpretations of *we both* and *we all* do, in fact, obtain.”

Coming back to children’s responses, I suspect that what is described in the discussion relating to quantifiers -- about how young children (don’t) understand quantifiers -- is mostly responsible for current findings, where the addition of “all” or “both” doesn’t reliably predict whether children pick one or more puppets to participate in a task. However, it might be worth taking a quick look at the number of additional puppets selected when they made group interpretations. If participants interpreted “we both” like I think I would (I would guess only four year olds would have this interpretation, if any children did), they should be more likely to select two versus three puppets for “we both” vs “we all.” This effect (if there is one) might be stronger in Study 2, since there are additional factors encouraging children to consider the other puppets as part of the speaker puppet’s group.

We thank the Reviewer for suggesting these insightful analyses that we had not considered previously. We conducted the analyses suggested. There was some evidence to suggest that the difference in the ratio of 3- to 4-puppet interpretations by 2-year-olds to the same ratio for 4-year-olds was greater in the second study than in the first study. Moreover, this was in the direction predicted by the Reviewer. Specifically, in Study 1, 2-year-olds chose 3-puppet group interpretations 22% of the time (*N* = 4) and 4-puppet group interpretations 78% of the time (*N* = 14). The same statistics for 4-year-olds were 26% (*N* = 7) and 74% (*N* = 20), respectively. Moreover, in Study 2, 2-year-olds chose 3-puppet group interpretations 17% of the time (*N* = 5) and 4-puppet group interpretations 83% of the time (*N* = 25). The same statistics for 4-year-olds were 33% (*N* = 16) and 67% (*N* = 32), respectively. However, several models suggested that none of these were reliable statistical differences nor that they varied reliably depending on whether children heard “we both” or “we all.” We have not included discussion of this point in the revised manuscript, but we have retained the code in the associated programming script for interested readers.

For future, I think using an in-person version of this would help as it would eliminate the confounding variable where some people are in one physical space and others are not. I also suggest including unambiguous pronominal phrases like “you and I,” “you,” and “I” to ensure that participants respond to those phrases as expected. This way, when surprising interpretations of “we”, “we both” and/or “we all” are discovered, they can be more assuredly tied to children’s interpretations of these phrases, rather than their confusion about how *any* pronoun works in the current context.

We kindly point the Reviewer to our second and third comments, above, which respond, respectively, to their comments that (i) “using an in-person version… others are not” and that (ii) “I also suggest… phrases as expected.”

Two additional comments/questions:

The inclusion of sex as a predictor variable is not motivated in the introduction/hypotheses.

We have revised the Introduction Section to better motivate the inclusion of sex as a predictor in the models reported in the studies in the manuscript. Specifically, in the revised Introduction Section (p. 6), we note that “Both studies also investigated effects of sex on interpretation of *we*. Previous studies of pronoun development suggest that males may form group interpretations of *we* more often than females (Vasil et al., 2024)”

What steps were taken to ensure that children were typically developing (or at least typically developing, with respect to language development)?

We have revised the Methods Sections of Studies 1 and 2 to clarify the steps taken to ensure that children were typically developing. Our recruitment processes for virtual studies only contact caregivers of participants who are typically developing, as per a statement made by caregivers prior to their child’s participation. We note this in the revised manuscript on pp. 7 and 21 by stating that “Participants… were typically developing (per caregiver statement).
